# Unfolded Protein Response Differentially Regulates TLR4-Induced Cytokine Expression in Distinct Macrophage Populations

**DOI:** 10.3389/fimmu.2019.01390

**Published:** 2019-06-21

**Authors:** Lei Zhang, Paul G. Pavicic, Shyamasree Datta, Qiaoling Song, Xiaohan Xu, Wei Wei, Fan Su, Patricia A. Rayman, Chenyang Zhao, Thomas Hamilton

**Affiliations:** ^1^School of Medicine and Pharmacy, Ocean University of China, Qingdao, China; ^2^Department of Inflammation and Immunity, Lerner Research Institute, Cleveland Clinic Foundation, Cleveland, OH, United States; ^3^School of Life Science, Lanzhou University, Lanzhou, China

**Keywords:** unfolded protein response, macrophage, cytokine, liver injury, toll like receptor

## Abstract

Cellular stress responses are often engaged at sites of inflammation and can alter macrophage cytokine production. We now report that macrophages in distinct states of differentiation or in different temporal stages of inflammatory response exhibit differential sensitivity to cell stress mediated alterations in M1-like polarized inflammatory cytokine production. Tunicamycin (Tm) treatment of bone marrow derived macrophages (BMDM) cultured with M-CSF cultured bone marrow derived macrophages (M-BMDM) had markedly amplified M1-like responses to LPS, exhibiting higher levels of IL12p40 and IL12p35 mRNAs while BMDM cultured with GM-CSF, which normally express high IL12 subunit production in response to LPS, were relatively unaltered. Anti-inflammatory IL10 mRNA production in LPS-stimulated M-BMDM was greatly reduced by cell stress. These changes in cytokine mRNA levels resulted from altered rates of transcription and mRNA decay. Stress also altered cytokine protein production. Resident liver macrophages isolated from mice treated with Tm showed elevated levels of IL12 subunit mRNA production following LPS stimulation. Furthermore, macrophages infiltrating the liver during the early phase of acetaminophen injury (24 h) had little stress-mediated change in cytokine mRNA production while cells isolated in the later phase (48–72 h) exhibited higher sensitivity for stress elevated cytokine production. Hence cultured macrophages developed using different growth/differentiation factors and macrophages from different temporal stages of injury *in vivo* show markedly different sensitivity to cell stress for altered inflammatory cytokine production. These findings suggest that cellular stress can be an important modulator of the magnitude and character of myeloid inflammatory activity.

## Introduction

It is now well-recognized that phenotypic heterogeneity or plasticity within myeloid cells is an important feature in the pathogenesis of many acute and chronic disorders (1–3). Macrophage lineage cell populations exhibit a broad array of activities in response to prototypic stimuli encountered in their local tissue microenvironment. Though such phenotypes are highly diverse they cover a range defined by two polarized endpoints, named classically activated M1 with potent pro-inflammatory features, or alternatively activated M2 with reparative, anti-inflammatory features (4–6). It is worth noting that most macrophages *in vivo* do not exhibit hyper-polarized M1 or M2 phenotype and indeed may exhibit M1 and M2 characteristics simultaneously. During response to injury or infection, the state of macrophage polarization has been shown to transition from M1 to M2 based upon the nature of the inflammatory environment and reflecting the change from inflammatory anti-microbial activity to the need for tissue restorative function (1, 7, 8). However, the magnitude of M1 or M2 like responses can vary dramatically.

For example, macrophages generated *in vitro* from bone marrow progenitors driven by the myeloid colony stimulating factors granulocyte-macrophage colony stimulating factor (GM-CSF) or macrophage colony-stimulating factor (M-CSF) are known to exhibit differences in magnitude, duration, and character of response to polarizing stimuli including LPS in both mice and humans (9–14). Although both factors were originally identified as factors promoting the growth and differentiation of myeloid progenitors, their role in regulating the magnitude of macrophage responses to polarizing stimuli is increasingly appreciated (15–19). Indeed, M-CSF and GM-CSF likely contribute differentially to the functional capacity of specific subsets of macrophages (13, 15, 20). M-CSF is constitutively expressed in almost all tissues and may modulate the nature of M1 and M2 responses in resident macrophages and in infiltrating monocytes. In contrast, enhanced M1 responses may be driven by locally elevated expression of GM-CSF seen during prolonged inflammatory processes (10, 20).

Cellular stress responses result from disruption of cellular homeostasis by microenvironmental challenges and serve to repair damage and restore homeostasis (21, 22). The unfolded protein response (UPR), also called endoplasmic reticulum (ER) stress, is an evolutionarily conserved complex set of pathways triggered by accumulation of unfolded or misfolded proteins in the lumen of the ER. Different conditions including nutrient deprivation, hypoxia, UV radiation, etc., can engage UPR-related stress response pathways in order to restore normal function by halting global protein translation, degrading misfolded proteins, and increasing the expression of molecular chaperones involved in protein folding. Conditions at sites of inflammation are likely to promote cell stress with substantial impact on many different aspects of the immune response (23–29). Moreover, cell stress and inflammation often occur in the same space and time (30–32) and engagement of cell stress during inflammatory TLR signaling leads to enhanced expression of a select subset of cytokine genes (28, 29, 33–37). Direct connections between cellular stress pathways and TLR responses have been previously reported. For example, the UPR has been linked to the activation of NFκB through IRE-1 and eIF2α and TLRs have been shown to activate components of the UPR (33, 36, 38). However, whether the status of macrophage differentiation influences sensitivity for stress-mediated change in TLR-induced cytokine production is not known.

In the present study, we found that environmental stress can dramatically amplify TLR4-stimulated IL12 subunit mRNA expression in M-CSF cultured bone marrow derived macrophages (M-BMDM) but not in GM-CSF cultured macrophage populations (GM-BMDM). Interestingly, engaging the UPR also selectively reduces anti-inflammatory cytokine IL10 in M-BMDM, thus altering the character and magnitude of the macrophage M1 polarized phenotype. The changes in cytokine mRNA levels require regulation in both transcriptional and post-transcriptional processes and depend upon the TLR4 adaptor protein TRIF. Finally, it is recognized that cell culture models do not accurately represent functional polarization as it operates *in vivo*. Hence we confirmed the effects of cell stress exposure on cytokine expression potential using several populations of macrophages obtained from the liver both with and without inflammatory challenge.

## Materials and Methods

### Reagents

ACK (Ammonium-Chloride-Potassium) lysis buffer, DMEM, and FBS were purchased from GIBCO, PBS was obtained from Biosharp, and Hyclone was the source of antibiotics. Tri-Reagent was purchased from Molecular Research Center and Immobilon-P transfer membrane was purchased from Thermo Fisher Scientific. RNase inhibitor, collagenase D and SYBR Green PCR Master Mix (2×), phosphatase and protease inhibitors cocktails were from Roche, PrimeScriptTM RT reagent kit with gDNA eraser was from TaKaRa. Acetaminophen (APAP), actinomycin D (Act D), LPS, and Histodenz were obtained from Sigma-Aldrich. Tunicamycin (Tm), cell lysis buffer (9803s), the antibody against GAPDH (clone 14C10), the antibody against caspase 3 and cleaved caspase 3 (clone 8G10) were purchased from Cell Signaling Technology. TGFβ, IL10, and IL12p70 ELISA kit, recombinant mouse M-CSF and GM-CSF were purchased from R&D Systems. Anti-CD11b-PE (clone M/70), anti-F4/80-FITC (clone BM8) were obtained from eBioscience. Anti-Ly6C-AlexaFluor (clone ER-MP20) was obtained from Biorad. Anti-CD206-PE/Cy7 (clone C068C2) was from Biolegend. Anti-PE microbeads were obtained from Miltenyi Biotec. Alanine Transaminase Activity (ALT) Assay Kit was from Nanjing Jiancheng Bioengineering. The peroxidase-conjugated affinipure goat anti-rabbit IgG was obtained from Jackson Immuno Research. Chemiluminescent HRP substrate was purchased from Millipore. BCA protein assay kit, Hochest 33342 (cat. No. C0030), and PI (cat. No. C0080) solution were from Solarbio Life Sciences. Annexin V-FITC Apoptosis detection Kit was from eBioscience.

### Mice and Bone Marrow Derived Macrophage Culture

C57BL/6 wild type mice and TRIF-/- mice on C57BL/6 background were obtained from The Jackson Laboratory and maintained in a temperature- and humidity- controlled room with a 12-h light-dark cycle. Mouse BMDMs were prepared by collecting the bone marrow cells from femurs. The cell suspensions were passed through a 100-micron nylon cell strainer (BD Falcon), collected by centrifugation at 300 g for 10 min, and resuspended in DMEM containing 10% FBS, 100 U/ml penicillin, 100 mg/ml streptomycin, either with 50 ng/ml M-CSF or 20 ng/ml GM-CSF. Cells (5 × 10^6^ for M-CSF or 10 × 10^6^ for GM-CSF) were seeded in a 100-mm dishes and cultured at 37°C with 5% CO_2_ for 7 days. Non-adherent cells were removed by washing, and the BMDMs were treated with DMSO or Tm (1 μg/ml) for 6 h prior to stimulation with LPS (100 ng/ml) for the indicated times as described in the text. All animal experiments were approved by the Cleveland Clinic Foundation Institutional Animal Care and Use Committee (IACUC).

### Reverse Transcription and Real Time PCR

Total RNA was prepared using Tri Reagent and treated with genomic eraser from PrimeScript^TM^ RT reagent kit to remove the possibly contaminated genomic DNA. One microgram DNA-free total RNA was used to make cDNAs following the instructions of the PrimeScript^TM^ RT reagent kit. The real-time PCR procedure were described previously (38). The specificity of the amplification products was confirmed by melting curve. The specific gene expression levels were determined by ΔΔCt method and individual values for specific genes were normalized to the ΔΔCt value of β-actin. Specific primers used in the real-time PCR to amplify corresponding mRNAs are as follows: IL12p35 forward, 5′-CTGTGCCTTGGTAGCATCTAT-3′ and reverse, 5′- CAGAGTCTCGCCATTATGATT−3′; IL12p40 forward, 5′- AGGTGCGTTCCTCGTAGAGA−3′ and reverse, 5′-AAAGCCAACCAAGCAGAAGA-3′; IL10 forward, 5′-CAGGGATCTTTAGCTAACGGAA-3′ and reverse, 5′-GCTCAGTGAATAAATAGAATGGGAAC-3′. The primers for primary transcript determination are: IL12p35 forward, 5′-CTCACTCCTCTGCTGCCAAA−3′ and reverse, 5′-TACGCGGGGACTGTCTCTTA-3′; IL12p40 forward, 5′-AGTGACATGTGGAATGGCGT-3′ and reverse, 5′-ACGTGGCAGACATCGTCTTT-3′; IL10 forward, 5′-AGTACAGCCGGGAAGACAATAA-3′ and reverse, 5′-GAAGGAGGAGGAAGAGAAGGAG-3′.

### Measurements of Primary Transcripts and mRNA Decay

M-BMDM or GM-BMDM cells were cultured as described above. On Day 8, BMDMs were treated with DMSO or Tm for 6 h, then followed by LPS treatment for the indicated times. Mature mRNA levels were determined using the primers indicated above and data are presented as the ratio of specific mRNA normalized to β-actin mRNA in each sample. For measurement of primary transcripts, total RNA was isolated and amplified using primers that span an intron exon junction. These primers will not amplify mature RNAs as they do not contain intron sequences. For measurement of mRNA half-life, the detailed methodology was descripted previously (39–41). Briefly, 8th day cultured BMDM were exposed to stress or not, then treated with LPS for 1 h. Actinomycin D was added to terminate transcription and after the indicated times, total RNA was prepared and used to determine remaining specific mRNA by real-time PCR using primers to amplify mature mRNA. Primary transcripts are not included in this measurement because the intron containing sequences are too long to be amplified during the short PCR amplification time and the melting curves show only a single PCR product. IL12p35, IL12p40, and IL10 mRNA levels were normalized to levels of β-actin mRNA measured in the same RNA sample. In each experiment, specific mRNA levels at zero time were arbitrarily assigned a value of 100% and remaining mRNA were determined relative to this. The data were used to obtain a best fit linear solution in a semi-log plot, and the equation obtained for each data set was then used to calculate the half-life. Values for half-life from 3 separate individual experiments were used to determine the mean ± S.D.

### Western Blot and Elisa

The cells were harvested and lysed on ice in cell lysis buffer (Cell Signaling Technology) in the presence of protease and phosphatase inhibitors (Roche). The protein concentrations were measured by BCA protein assay kit (Solarbio). The proteins were separated by SDS-PAGE, transferred to nitrocellulose blotting membranes, blocked with 5% non-fat milk, 0.1% Tween-20 in Tris buffered saline (TBS), and incubated with caspase 3 and GAPDH primary antibodies. Horseradish peroxide-conjugated secondary antibodies were used, and the proteins were detected using ECL Detection Reagent and photographed by Tanon 5200 imaging system. The IL12p70, IL10, and TGFβ levels in the supernatant of BMDMs were measured using commercial ELISA kits (R&D Systems) following the user instructions.

### Acetaminophen (APAP) Induced Liver Injury

APAP was dissolved in warmed PBS at 7 mg/ml. Six to eight weeks old C57BL/6 male mice were fasted overnight and weighed prior to intraperitoneal injection of APAP (300 mg/kg body weight). Access to food was restored 30 min after APAP administration.

### *In vivo* Tunicamycin (Tm) Injection

Tm was dissolved in DMSO at 10 mg/ml. Six to eight weeks old C57BL/6 male mice were challenged (i.p.) with DMSO or Tm (1.25 mg/kg body weight) for 18 h prior to the isolation of non-parenchymal cells from the liver. In APAP induced liver injury model, Tm or DMSO were given (i.p.) 18 h before harvest.

### Preparation of Liver CD11b+ Cells

Non-parenchymal cells were isolated from normal male mouse liver or from liver obtained from APAP challenged mice and prepared as described previously (38) with minor modifications. Briefly, the liver was perfused with D-PBS solution via the inferior vena cava, minced with Gentlemacs tissue dissociator (Miltenyi Biotec) and digested with collagenase D (1 mg/ml) for 20 min at 37°C with agitation. The digests were filtered through 100 μm cell strainer, washed with D-PBS for several times and the non-parenchymal cells were enriched by 30% Histodenz density gradient centrifugation. A portion of the non-parenchymal cells were then immunostained with anti-CD11b-PE, anti-Ly6C-AlexaFluor, and anti-F4/80-FITC antibody for flow cytometry analysis. The remaining non-parenchymal cells were labeled with anti-CD11b-PE antibody followed by incubation with anti-PE magnetic microbeads. CD11b+ cells were collected by passing antibody and microbeads conjugated non-parenchymal cells through the MACS separation columns in the magnetic field.

### Flow Cytometry

The non-parenchymal cells obtained from the Histodenz density gradient centrifugation were washed with PBS and resuspended in FACS buffer (2% FBS and 0.2% sodium azide in PBS). After blocking, the cells were immunostained with anti-CD11b, anti-Ly6C, anti-F4/80, and CD206 antibodies. Titrated concentrations were used and stained cell samples were examined on a BD LSRFortessa flow cytometer using FACSDiva v8.0.1. All analyses were performed using FlowJo 10.4 software. Fluorescence minus one (FMO) controls were used to determine the background levels of staining and adjust multicolor compensation as gating strategy.

### Cell Viability Measurements

BMDMs were pretreated with Tm (1 mg/ml) for 6 h prior to stimulation with LPS (100 ng/ml) 10 h. Cell apoptosis was measured by an Annexin V staining kit according to the manufacturer's instructions (eBioscience). Briefly, BMDMs in non-TC (tissue culture) treated dishes were detached by trypsinization and washed with PBS. Cells were resuspended in binding buffer and stained with Annexin-V-FITC solution. The proportion of Annexin V+ cells was calculated in the gated single cell population by BD LSRortessa flow cytometer with FlowJo software. The average fluorescence intensity of the Annexin V+ cells in each group was obtained in the gated Annexin V+ cell population and analyzed by FlowJo software. For PI immunofluorescence microscopy assay, the cells were washed with cold PBS and incubated with Hochest 33342 (10 μg/ml) and PI (1 mg/ml) solution (Solarbio) for 15 min. The cells were observed under Zeiss Vert.A1 microscope. Fluorescence images were captured with Zeiss Axiocam 503 color CCD camera controlled with ZEN software (ZEISS).

### Statistics

All the data are presented as mean ± SD. The homogeneity of variance was evaluated by Levene test in SSPE. Two-way ANOVA followed by Tukey's multiple comparisons test were performed to evaluate the difference between treatment groups using GraphPad Prism version 7 software. Differences with *p* < 0.05 were considered significant.

## Results

### Cellular Stress Amplifies LPS-Induced IL12 Gene Expression in M-BMDM but not GM-BMDM

**Figure 1:**
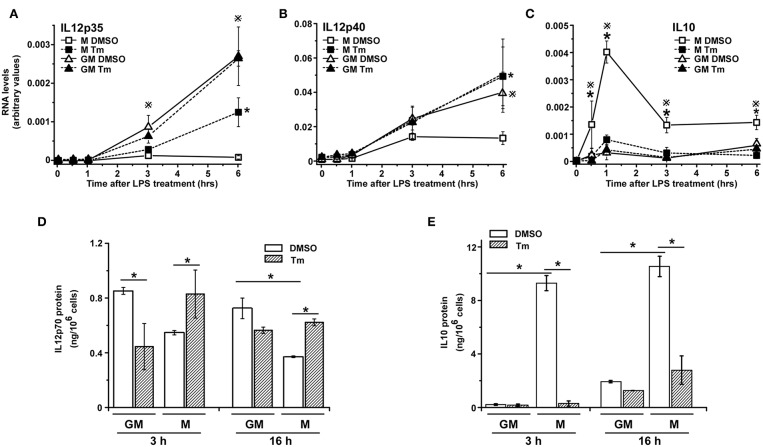
Cellular stress modifies cytokine expression in BMDM. **(A–C)** M-BMDM and GM-BMDM were treated with DMSO or Tm (1 μg/ml) for 6 h prior to stimulation with LPS (100 ng/ml) for the indicated times. Levels of IL12p35 **(A)**, IL12p40 **(B)**, or IL10 **(C)** mRNA levels were determined by real time PCR and are presented as described in Materials and Methods. **(D,E)** M-BMDM and GM-BMDM were treated with DMSO or Tm for 6 h followed by LPS for 3 h. The cells were washed 3 times with warmed PBS, fresh medium was added and incubation continued for another 3 or 16 h. Supernatants were collected and used to determine protein levels of IL12p70 **(D)** and IL10 **(E)** by ELISA. Data are presented as the mean ± SD for triplicate experiments and the differences between the indicated treatments were evaluated by two way-ANOVA and Turkey's multiple comparisons test. In **(A–C)**, *P* < 0.05 is indicated by * for comparison of stress vs. DMSO in M-BMDMs, by # for comparison of stress vs. DMSO in GM-BMDMs, by ※ for comparison of M DMSO vs. GM DMSO and by Ψ for comparison of M-BMDM stress vs. GM-BMDM DMSO. In **(D,E)**, *P* < 0.05 is indicated by * for comparison of the indicated groups.

We have previously reported that the pattern of inflammatory chemokine CXCL1 gene expression in LPS-stimulated M-BMDM is modified by coincident cellular stress (38). Because macrophages developed using different growth/differentiation factors (M-CSF and GM-CSF) exhibit different responses to M1 polarizing stimuli (42, 43), we began to explore whether exposure to cell stress might alter TLR-induced cytokine expression differentially depending upon the state of differentiation. Though both populations are CD11b+, M-BMDM are highly enriched for the resident tissue macrophage marker F4/80 while GM-BMDM more closely resemble the inflammatory monocytes and exhibit lower levels of F4/80 and higher levels of LY6C (Figure S1A). We have used this model to evaluate the effect of tunicamycin (Tm)-induced cell stress on expression of the LPS-stimulated prototypic M1 cytokine IL12. BMDMs cultured with either M-CSF (M-BMDM) or GM-CSF (GM-BMDM) were treated with Tm for 6 h to induce cell stress. Cell stress did not significantly alter the levels of TGFβ (Figure S1B). The LPS treatment time was limited to 6 h as stress-mediated apoptosis increased substantially at later times (Figure S2). The 6 h pretreatment period was also selected based upon the time course for expression of cellular stress markers (CHOP, BIP, spliced XBP1; see Figure S1C). When unstressed cells were treated with LPS for different times, cultures of GM-BMDM exhibited substantially greater expression of mRNA encoding both IL12 subunits (IL12p35 and IL12p40) than did M-BMDM cultures (Figures 1A,B). When treated with Tm, however, the level of IL12 subunit mRNA expression in M-BMDM was markedly elevated while there was little or no effect of stress on response to LPS in GM-BMDM. The levels of secreted IL12p70 protein are also selectively increased in M-BMDM (Figure 1D). The opposite behavior was observed when expression of the anti-inflammatory cytokine IL10 was measured (Figures 1C,E). While unstressed M-BMDM produced the highest level of IL10 mRNA, this was dramatically reduced by Tm-induced stress. GM-BMDM produced little IL10 mRNA in either condition. Moreover, the capacity of cellular stress to modulate cytokine gene expression was not limited to IL12 and IL10 as similar observations were made when TNFα, CXCL1, and IL6 mRNA levels were measured (Figures S3A–C). Hence it appears that stress modulates prototypic M1-like cytokine expression in M-BMDM but has little or no effect on M1 like response in GM-BMDM.

### Cellular Stress Modulates Cytokine Expression by Altering Transcription and mRNA Half Life

**Figure 2:**
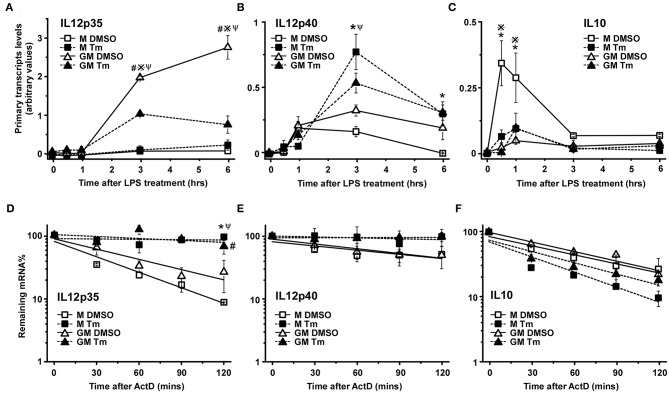
Cellular stress modifies cytokine mRNA expression at both transcriptional and post-transcriptional levels. **(A–C)** M-BMDM and GM-BMDM were treated with DMSO or Tm for 6 h prior to stimulation with LPS for the indicated times and total RNA was used to determine primary transcript levels of IL12p35 **(A)**, IL12p40 **(B)**, or IL10 **(C)** by real time PCR as described in Materials and Methods. **(D–F)** M-BMDM and GM-BMDM were treated with DMSO or Tm for 6 h and LPS for 1 h prior to addition of actinomycin D (Act D, 5 μg/ml) for indicated times. The remaining IL12p35 **(D)** IL12p40; **(E)** or IL10 **(F)** mRNA levels were determined by real time PCR as described in Materials and Methods. Data are presented as the mean ± SD for triplicate experiments and the differences between the indicated treatments were evaluated by two way-ANOVA and Turkey's multiple comparisons test. *P* < 0.05 is indicated by * for comparison of stress vs. DMSO in M-BMDMs, by # for comparison of stress vs. DMSO in GM-BMDMs, by ※ for comparison of M DMSO vs. GM DMSO and by Ψ for comparison of M-BMDM stress vs. GM-BMDM DMSO.

Cytokine gene expression is often transient and as such is subject to regulation through modulation of both transcription and mRNA half-life (44). To evaluate the mechanistic basis for the effects of stress on cytokine gene expression in the different cell populations we have measured the impact of Tm-induced stress on transcription and mRNA decay for IL12p35, IL12p40, and IL10 in TLR4-stimulated M-BMDM and GM-BMDM. Transcription was assessed by measuring primary transcript abundance using primers that span an intron exon junction (found only in transcripts and not in mature mRNA) while mRNA half-life was estimated by following the decay of mature mRNAs over time following addition of the transcriptional inhibitor Actinomycin D (Act D). At least 3 distinct patterns contribute to the stress-mediated change in levels of mRNA encoding the three different cytokines. Though Tm resulted in a substantial increase in IL12p35 mRNA levels in M-BMDM (Figure 1A), there was almost no change in primary transcript abundance (Figure 2A). Rather, IL12p35 mRNA was markedly stabilized in M-BMDM (Figure 2D). In GM-BMDM, however, Tm treatment markedly reduced transcription but increased mRNA half-life and hence there was little change in IL12p35 mRNA levels in this cell population (Figures 2A,D). Primary transcript levels for IL12p40 were markedly elevated in Tm-treated M-BMDM but were unchanged in GM-BMDM (Figure 2B). Though stress elevated the level of both IL12p35 and IL12p40 mRNAs in M-BMDM populations (Figures 1A,B), the mechanisms were quite different for IL12p40 vs. IL12p35 (Figures 2A,B,D,E). Thus, IL12p40 appears to be controlled primarily by changing transcription while changes in IL12p35 mRNA involve prolongation of half-life. Finally, Tm-treatment markedly decreased the abundance of IL10 primary transcripts and had little impact on half-life (Figures 2C,F).

### The Effects of Stress on TLR4 Signaling Requires TRIF

**Figure 3:**
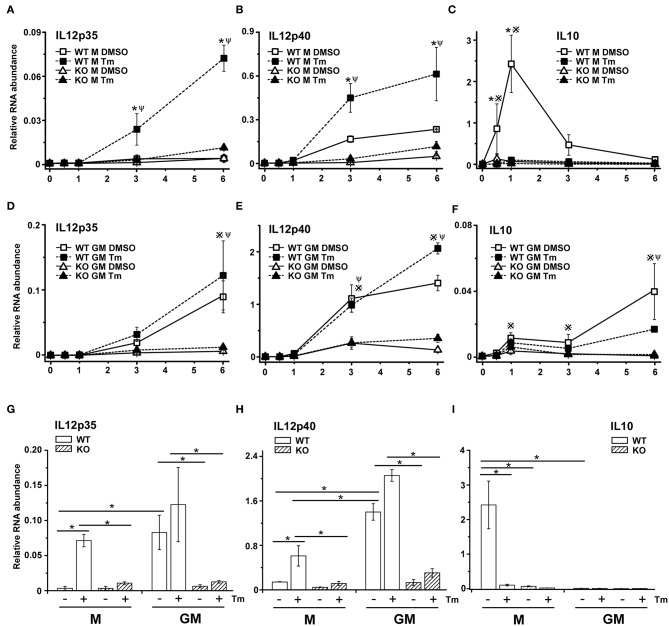
The adaptor protein TRIF is necessary for stress-mediated change in TLR4-dependent cytokine mRNA expression. M-BMDM **(A–C)** or GM-BMDM **(D–F)** from wild type and TRIF-/- mice were treated with DMSO or Tm for 6 h followed by LPS for the indicated times. Total RNA was prepared and used to determine IL12p35 **(A,D)**, IL12p40 **(B,E)**, or IL10 **(C,F)** mRNA levels by real time PCR. The mRNA levels of IL12p35 **(G)** and IL12p40 **(H)** after LPS stimulation for 6 h and IL-10 after LPS stimulation for 1 h **(I)** were compared between each group, respectively. Data are presented as the mean ± SD of triplicate experiments and the differences between indicated treatments were evaluated by two way-ANOVA and Turkey's multiple comparisons test. In **(A–F)**, *P* < 0.05 is indicated by * for comparison of DMSO vs. stress in WT BMDMs, by # for comparison of DMSO vs. stress in TRIF-/- BMDMs, by ※ for comparison of WT DMSO vs. TRIF-/- DMSO and by Ψ for comparison of WT stress vs. TRIF-/- stress. In **(G–I)**, *P* < 0.05 is indicated by * for comparison of the indicated groups.

We previously reported that the amplified expression of CXCL1 mRNA in stressed BMDM in response to TLR4 stimulation was dependent on the signaling adaptor protein TRIF (38). To extend this observation to IL12 and determine if the differential stress sensitivity in M-BMDM vs. GM-BMDM was dependent upon TRIF, we examined IL12p35, IL12p40, and IL10 mRNA levels in stressed M-BMDMs and GM-BMDMs prepared from wildtype and TRIF-deficient mice. Expression of both IL12 subunits were dramatically dependent upon the presence of TRIF (Figures 3A,B,D,E,G,H). The ability of stress to amplify LPS-induced p35 and p40 expression in M-BMDMs from TRIF-/- was fully compromised (Figures 3A,B,G,H). Interestingly, the elevated level of both IL12 mRNA subunits seen in GM-BMDMs was markedly reduced in cultures prepared from TRIF-/- mice (Figures 3D,E,G,H). This suggests that the signals through which stress modulates the response to LPS in M-BMDM and GM-BMDM share substantial similarity. Moreover, TRIF deficiency diminished the induction of IL10 expression in M-BMDM (Figures 3C,F,I), supporting the critical role for TRIF in TLR4-induced IL10 previously reported (45, 46).

### Liver Resident Macrophages Exhibit Stress-Sensitive Cytokine mRNA Expression

**Figure 4:**
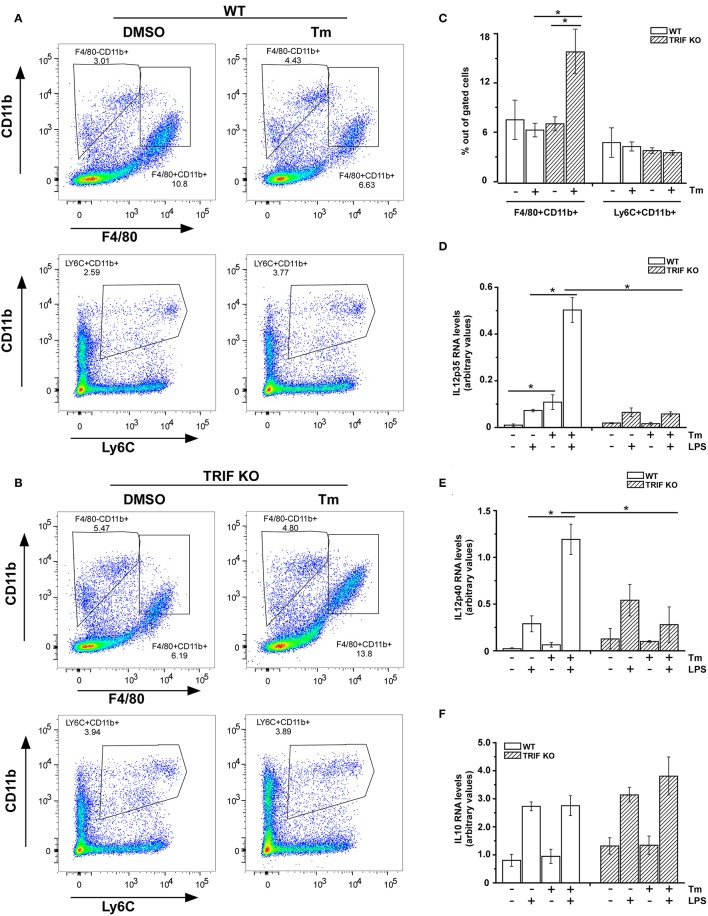
Resident macrophages from liver are sensitive to stress-induced change in cytokine mRNA expression. **(A,B)** WT **(A)** and TRIF-/- **(B)** mice were challenged with DMSO or Tm (1.25 mg/kg body weight) for 18 h prior to the isolation of non-parenchymal cells from the liver. The non-parenchymal cells were immunostained for surface CD11b, Ly6C, and F4/80 and subjected to flow cytometry analysis. **(C)** The percentage of LY6C+ and F4/80+ cells within the gated populations isolated from livers of mice treated with DMSO vs. Tm (*n* = 4). **(D–F)** Liver non-parenchymal cells from WT or TRIF-/- mice treated with DMSO or Tm were labeled with anti-CD11b-PE antibody and then isolated using anti-PE magnetic microbeads. CD11b+ cells were subsequently treated with or without LPS (100 ng/ml) for 3 h. Total RNA was used to determine IL12p35 **(D)**, IL12p40 **(E)**, and IL10 **(F)** mRNAs by real time PCR. Data are presented as the mean ± SD of triplicate experiments for WT (*n* = 3 per treatment for each experiments) and duplicate experiments for TRIF-/- mice (*n* = 3 per treatment for each experiments). The differences between indicated treated samples were evaluated by two way-ANOVA and Turkey's multiple comparisons test. *p < 0.05.

We appreciate that *in vitro* cultured BMDM are unlikely to accurately reflect the status of macrophage populations within tissues at rest or following injury. To address the question of whether populations of tissue macrophages would exhibit the modulation of cytokine gene expression under conditions that activate cellular stress responses, we examined cytokine gene expression in macrophages prepared from mice subjected to stress *in vivo* by i p. injection of Tm. As a first step we compared resident macrophages prepared from the liver in mice receiving either PBS or Tm (25 μg/mouse) 24 h before sacrifice. CD11b, F480, and Ly6C expression patterns in non-parenchymal cells prepared from the livers of WT mice treated with or without Tm were not markedly different from one another and resembled resident liver macrophage populations, based upon expression of F4/80 and LY6C (Figures 4A,C). When these cells were cultured and treated with LPS *in vitro* for 3 h, cells from Tm-pretreated mice exhibited elevated expression of IL12p35 and p40 mRNAs as compared to cells obtained from vehicle treated mice (Figures 4D,E). There was little effect of Tm-mediated stress on the expression of IL10 in response to LPS treatment (Figure 4F). We next asked if stress-induced amplification of IL12 subunit mRNA expression *in vivo* is also mechanistically dependent upon signaling through TRIF. As in WT resident liver macrophages, the surface expression of Ly6C were not altered by stress pretreatment, however, the expression of CD11b in this population is increased (Figures 4B,C). The levels of LPS-induced IL12p35 and IL12p40 mRNA, however, were not different between macrophages from Tm treated and untreated TRIF-/- mice (Figures 4D,E) indicating the important regulatory role of TRIF in stress enhanced LPS-induced IL12 expression in resident liver macrophages. Consistent with cell culture results, the expression of IL10 was comparable in both WT and TRIF-/- resident liver macrophages (Figure 4F).

### Stress Sensitivity for Inflammatory Cytokine Expression in Infiltrating Inflammatory Macrophages

**Figure 5:**
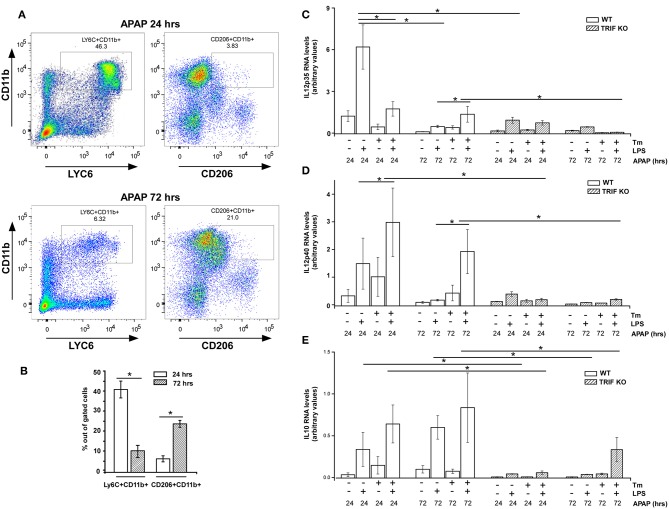
Sensitivity to stress-induced modulation of cytokine expression in infiltrating liver myeloid cells varies over the course of inflammatory response. **(A)** WT Mice were injected i.p. with APAP (300 mg/kg body weight) for 24 or 72 h prior to the isolation of liver non-parenchymal cells. The isolated liver non-parenchymal cells were immunostained for surface CD11b, CD206, and Ly6C and analyzed by flow cytometry. **(B)** The percentage of LY6C+ and CD206+ cells within the gated cell populations (*n* = 4). **(C–E)** WT and TRIF-/- mice were injected i.p. with APAP (300 mg/kg) for 24 or 72 h and treated with DMSO or Tm i.p. during the final 18 h. Liver CD11b+ cells from WT and TRIF-/- mice were isolated using anti-CD11b-PE and MACS magnetic column followed by treatment with or without LPS (100 ng/ml) for 3 h. Total RNA was prepared and used to determine IL12p35 **(C)**, IL12p40 **(D)**, and IL10 **(E)** mRNA levels by real time PCR. Data are presented as the mean ± SD of triplicate experiments for WT (*n* = 3 per treatment for each experiments) and duplicate experiments for TRIF-/- mice (*n* = 3 per treatment for each experiments). The differences between indicated treatments were evaluated by two way-ANOVA and Turkey's multiple comparisons test. *p < 0.05 was considered significant.

It is well-recognized that response to injury is a dynamic process exhibiting infiltration by early proinflammatory myeloid cells followed by a transition of these cells to a reparative phenotype that contributes to restoration of tissue homeostasis (7, 8). Therefore, we wished to determine if the ability of cell stress to modulate inflammatory cytokine gene expression within macrophages might vary with the stage of the inflammatory process *in vivo*. This was tested by examining myeloid cells infiltrating the liver following acute injury due to acetaminophen (APAP) toxicity. At 24 h after APAP administration, tissue injury reaches a maximum as determined by release of liver enzymes (ALT) into the blood and this injury is largely repaired by 72 h, which demonstrated by both ALT levels as well as H&E staining (see Figures S4A,B). Following APAP injection, macrophages transition from a pro-inflammatory CD11b+/Ly6Chi/CD206lo phenotype at 24 h to CD11b+/Ly6Clo/CD206hi phenotype by 72 h (Figures 5A,B). CD11b+ macrophages prepared 24 h after APAP treatment were able to produce IL12p35 and p40 though Tm-induced stress did not modulate the IL12p35 response and had only modest effects on LPS-induced IL12p40 mRNA levels (Figures 5C,D). In contrast, cell populations obtained 72 h after injury have reduced levels of cytokine mRNA production which can be markedly amplified in animals receiving Tm during the 24 h period prior to harvest (Figures 5C,D). In both cases, cells from APAP-treated TRIF-/- mice did not exhibit sensitivity to Tm. These results are consistent with the differential sensitivity to cell stress seen in GM-BMDM and M-BMDM (Figure 1). Surprisingly, IL10 mRNA production was elevated in all WT cell populations and exhibited some modest elevation in those obtained from stressed animals (Figure 5E).

## Discussion

Plasticity is an important characteristic of mononuclear phagocytes that contributes to the heterogeneity of function within macrophage populations in different anatomic locations. Dysregulation of this process contributes to the pathogenesis of multiple disorders but our understanding of the regulatory features that control it remains limited. It is noteworthy that cellular stress pathways contribute to disease pathogenesis within multiple cell populations and indeed the modulation of macrophage function by cell stress response has been reported by multiple laboratories (22–28). We hypothesize that stress and inflammatory cytokine expression may couple differentially within specific myeloid cell subsets and play an important role in the magnitude, duration, and character of cytokine expression. Hence we have evaluated the effects of stress on IL12 and IL10 gene expression in macrophages exhibiting different phenotypes both *in vitro* using culture with GM-CSF or M-CSF and *in vivo* both in resident and infiltrating macrophages over the course of an inflammatory response. The results demonstrate that the lower capacity of M-CSF-cultured BMDM for IL12 subunit production can be markedly enhanced by cellular stress while the elevated expression of IL12 in GM-CSF BMDM is not effected. Anti-inflammatory IL10 expression exhibits the opposite behavior as coincident cell stress diminishes the elevated expression seen in M-BMDM but does not change the low expression seen in GM-BMDM. A similar pattern of behavior is observed when macrophage populations within the liver are evaluated for IL12 and IL10 expression following exposure to stress *in vivo*. Resident tissue macrophages prepared from the livers of mice treated with Tm to promote cell stress *in vivo* show elevated pro-inflammatory cytokine expression when stimulated through TLR4. Moreover, cell stress has little impact on cytokine expression in infiltrating pro-inflammatory macrophages from mice treated with APAP to induce acute liver injury. However, infiltrating macrophages prepared from mice during the recovery phase of the APAP injury show reduced cytokine expression that can be amplified if the mice are exposed to stress. Thus, stress appears to have very different effects on pro- and anti-inflammatory cytokine gene expression depending upon the status of the cells.

Historically, classically activated M1 and alternatively activated M2 macrophage phenotypes have been defined by the response to prototypic pro- and anti-inflammatory stimuli (IFNγ/LPS vs. IL4/IL10, respectively) (6, 8, 47, 48). However, the magnitude of response to these agents can vary substantially between different populations of macrophages. Indeed, macrophages generated by culture in the presence of the myeloid colony stimulating factors GM-CSF and M-CSF have been shown to exhibit responses that vary dramatically in the magnitude and duration of inflammatory cytokine production following stimulation with IFNγ and/or LPS; while GM-CSF promotes elevated M1 response to LPS stimulation, M-CSF favors a stronger M2-polarization status (14, 20). Although neither GM-CSF nor M-CSF are themselves potent stimuli of cytokine production when compared with responses to prototypic polarizing stimuli, they rather predispose the macrophages toward very different levels of pro-inflammatory or anti-inflammatory cytokine production when exposed to such stimuli. Thus in the current study, in response to LPS, GM-BMDM express high levels of IL12 but little IL10 while M-BMDM produce significant IL10 but little IL12. The current study extends these differences by demonstrating that these distinct states of differentiation produced by culture with the different CSF activities also exhibit quite different sensitivities to the effects of cell stress responses on the magnitude and duration of IL12 and IL10 cytokine production.

Stimulation of resident and infiltrating myeloid cells via TLRs is well-recognized to drive expression of inflammatory cytokines and the specificity, magnitude, and duration of TLR-initiated signaling events are important in determining the pathogenesis of multiple disorders (49). Furthermore, direct connections between cellular stress pathways and TLR responses have been previously reported. For example, engagement of the UPR can induce activation of NFκB via IRE-1 and eIF2α kinase function and TLRs have been shown to activate components of the UPR (33, 50–54). Interestingly, pretreatment with low doses of LPS could selectively attenuate the ATF4-CHOP branch of UPR pathway (55). Importantly, we previously showed that cell stress could markedly enhance the production of pro-inflammatory chemokine expression in M-BMDM (38). It is noteworthy that the stress enhancement depends upon the TLR4-specific adaptor protein TRIF. TLR4 signaling is known to utilize two pathways (MyD88 and TRIF) and the MyD88 component is clearly an important contributor to LPS-induced cytokine production. Furthermore, TRIF signaling is utilized by other TLRs (specifically TLR3) and responses to TLR3 are also known to exhibit stress sensitivity (38).

The changes in gene expression, for both IL12 subunits and for IL10 are largely reflected in levels of their specific mRNAs. While the changes result from alterations in gene transcription as well as mRNA half-life, these mechanisms are differentially operative for the 3 mRNAs. Of particular interest is the finding that both IL12p35 and p40 mRNAs are dramatically stabilized in stressed cells of both phenotypes. This is coincident with substantial changes in mRNA translation that are well-known consequences of cellular stress where the phosphorylation of eIF2α translational initiation factor blocks translational initiation of most mRNAs (28). This translational blockade could be mechanistically linked to the prolongation of mRNA half-life but it is evident that it operates in message specific fashion as the degradation of other mRNAs is not altered (38). The physiologic consequences of stress-mediated modulation of inflammatory response via prolongation of specific mRNA half-life and the associated increase in mRNA abundance might be advantageous by helping to compensate for the translational blockade by enabling continued cytokine production during the period of cell stress associated with inflammatory conditions in injured tissue. Furthermore, the ability for stress to increase pro-inflammatory capacity during the recovery phase of inflammatory response would enable maintenance of inflammatory cytokine production if conditions at the site change (e.g., new infection or injury).

## Ethics Statement

All animal experiments were approved by the Cleveland Clinic Foundation Institutional Animal Care and Use Committee (IACUC).

## Author Contributions

TH and CZ directed all aspects of the project and wrote the manuscript. LZ, PP, SD, QS, XX, WW, FS, and PR performed experiments. All authors reviewed the final manuscript.

### Conflict of Interest Statement

The authors declare that the research was conducted in the absence of any commercial or financial relationships that could be construed as a potential conflict of interest.

## Supplementary Material

Figure S1 Phenotypic characterization and stress activation in BMDM. **(A)** M-BMDM and GM-BMDM were immunostained for surface CD11b, F4/80, and Ly6C, then analyzed by flow cytometry. The percentage of F4/80+CD11b+ and Ly6C+CD11b+ cells in gated populations are presented. **(B)** M-BMDM and GM-BMDM were incubated with fresh media for 6 h, or BMDMs were treated with DMSO or Tm (1 μg/ml) for 6 h prior to stimulation with LPS (100 ng/ml) for 6 h. The supernatants were collected and the TGFβ protein levels were determined by ELISA. **(C)** M-BMDM and GM-BMDM were treated with DMSO or Tm (1 μg/ml) for 6 h prior to stimulation with LPS (100 ng/ml) for indicated times. Levels of CHOP, spliced XBP1 (sXBP1) and Bip mRNA were determined by real time PCR and presented as described in Materials and Methods. Values are the mean ± SD for triplicate experiments. The statistical analysis was performed by two-way ANOVA and Turkey's multiple comparisons test in Prism 7. In **(A,B)**, *P* < 0.05 is indicated by ^*^ for comparison of the indicated groups. In **(C)**, *P* < 0.05 is indicated by ^*^ for comparison of stress vs. DMSO in M-BMDMs, by # for comparison of stress vs. DMSO in GM-BMDMs, by ※ for comparison of M DMSO vs. GM DMSO and by Ψ for comparison of M-BMDM stress vs. GM-BMDM DMSO.

Figure S2 Cellular stress and TLR induced apoptosis in BMDMs. M-BMDM and GM-BMDM were treated with DMSO or Tm (1 mg/ml) for 6 h prior to stimulation with LPS (100 ng/ml) for 10 h. **(A)** Cells were stained for Annexin and analyzed by flow cytometry. The percentage of annexin V positive cells **(B)** and the mean fluorescence intensity **(C)** for each treatment group were quantified. **(D)** The cells were also stained with PI (red) and Hochest (blue). **(E)** Levels of cleaved caspase 3 protein from these cells were analyzed by Western blot. Data are presented as the mean ± SD of triplicate experiments and the differences between indicated treatments were evaluated by two way-ANOVA and Turkey's multiple comparisons test. *P* < 0.05 is indicated by ^*^ for comparison of the indicated groups.

Figure S3 Cellular stress amplifies TLR4 induce cytokine expression in BMDM. **(A–C)** M-BMDM and GM-BMDM were treated with DMSO or Tm (1 μg/ml) for 6 h prior to stimulation with LPS (100 ng/ml) for the indicated times. Levels of TNFα **(A)**, CXCL1 **(B)**, or IL6 **(C)** mRNA were determined by real time PCR and presented as described in Materials and Methods. Data are presented as the mean ± SD for triplicate experiments and the differences between DMSO and Tm treatments were evaluated by two way-ANOVA and Turkey's multiple comparisons test. *P* < 0.05 is indicated by ^*^ for comparison of stress vs. DMSO in M-BMDMs, by # for comparison of stress vs. DMSO in GM-BMDMs, by ※ for comparison of M DMSO vs. GM DMSO and by Ψ for comparison of M-BMDM stress vs. GM-BMDM DMSO.

Figure S4 Liver injury induced by APAP administration. **(A)** WT mice were injected i.p. with APAP (300 mg/kg) for 24 or 72 h and treated with DMSO or Tm i.p. during the final 18 h. The blood was collected for the measurement of ALT activity as described in Materials and Methods. **(B)** WT mice were treated with APAP only as in **(A)**, and the representative images of H&E-stained liver sections 24, 48, and 72 h post APAP challenge are shown (*n* = 5). Data are presented as the mean ± SD of triplicate experiments and the differences between indicated treatments were evaluated by two way-ANOVA and Turkey's multiple comparisons test. *P* < 0.05 is indicated by ^*^ for comparison of the indicated groups.
