# Supplementary figures and images for: Unfolded Protein Response Differentially Regulates TLR4-Induced Cytokine Expression in Distinct Macrophage Populations
Source: Front Immunol. 2019 Jun 21;10:1390. doi: 10.3389/fimmu.2019.01390 (PMC6598306; doi:10.3389/fimmu.2019.01390)

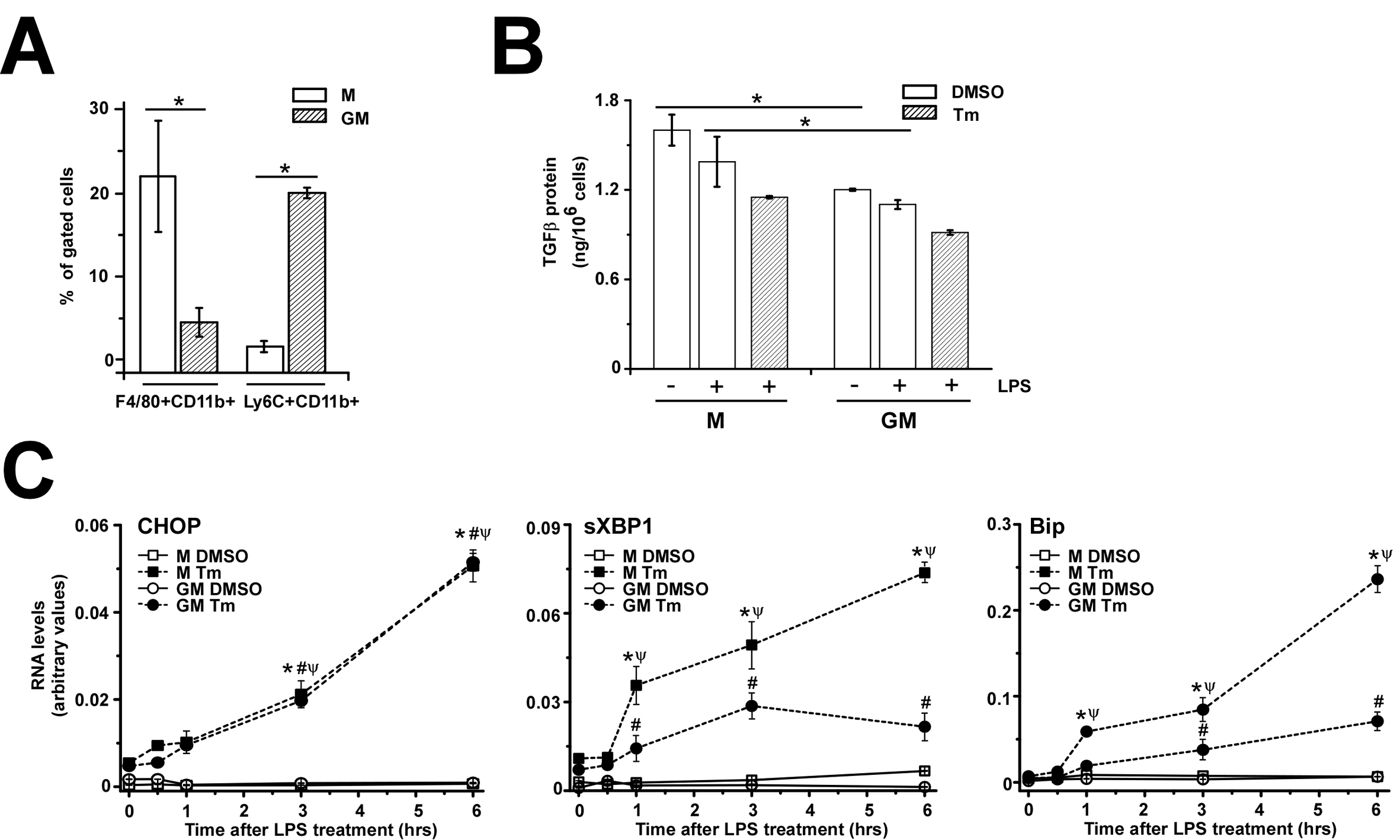

Supplement: Figure S1 — Phenotypic characterization and stress activation in BMDM. (A) M-BMDM and GM-BMDM were immunostained for surface CD11b, F4/80, and Ly6C, then analyzed by flow cytometry. The percentage of F4/80+CD11b+ and Ly6C+CD11b+ cells in gated populations are presented. (B) M-BMDM and GM-BMDM were incubated with fresh media for 6 h, or BMDMs were treated with DMSO or Tm (1 μg/ml) for 6 h prior to stimulation with LPS (100 ng/ml) for 6 h. The supernatants were collected and the TGFβ protein levels were determined by ELISA. (C) M-BMDM and GM-BMDM were treated with DMSO or Tm (1 μg/ml) for 6 h prior to stimulation with LPS (100 ng/ml) for indicated times. Levels of CHOP, spliced XBP1 (sXBP1) and Bip mRNA were determined by real time PCR and presented as described in Materials and Methods. Values are the mean ± SD for triplicate experiments. The statistical analysis was performed by two-way ANOVA and Turkey's multiple comparisons test in Prism 7. In (A,B), P < 0.05 is indicated by * for comparison of the indicated groups. In (C), P < 0.05 is indicated by * for comparison of stress vs. DMSO in M-BMDMs, by # for comparison of stress vs. DMSO in GM-BMDMs, by ※ for comparison of M DMSO vs. GM DMSO and by Ψ for comparison of M-BMDM stress vs. GM-BMDM DMSO. [file Image_1.TIF]

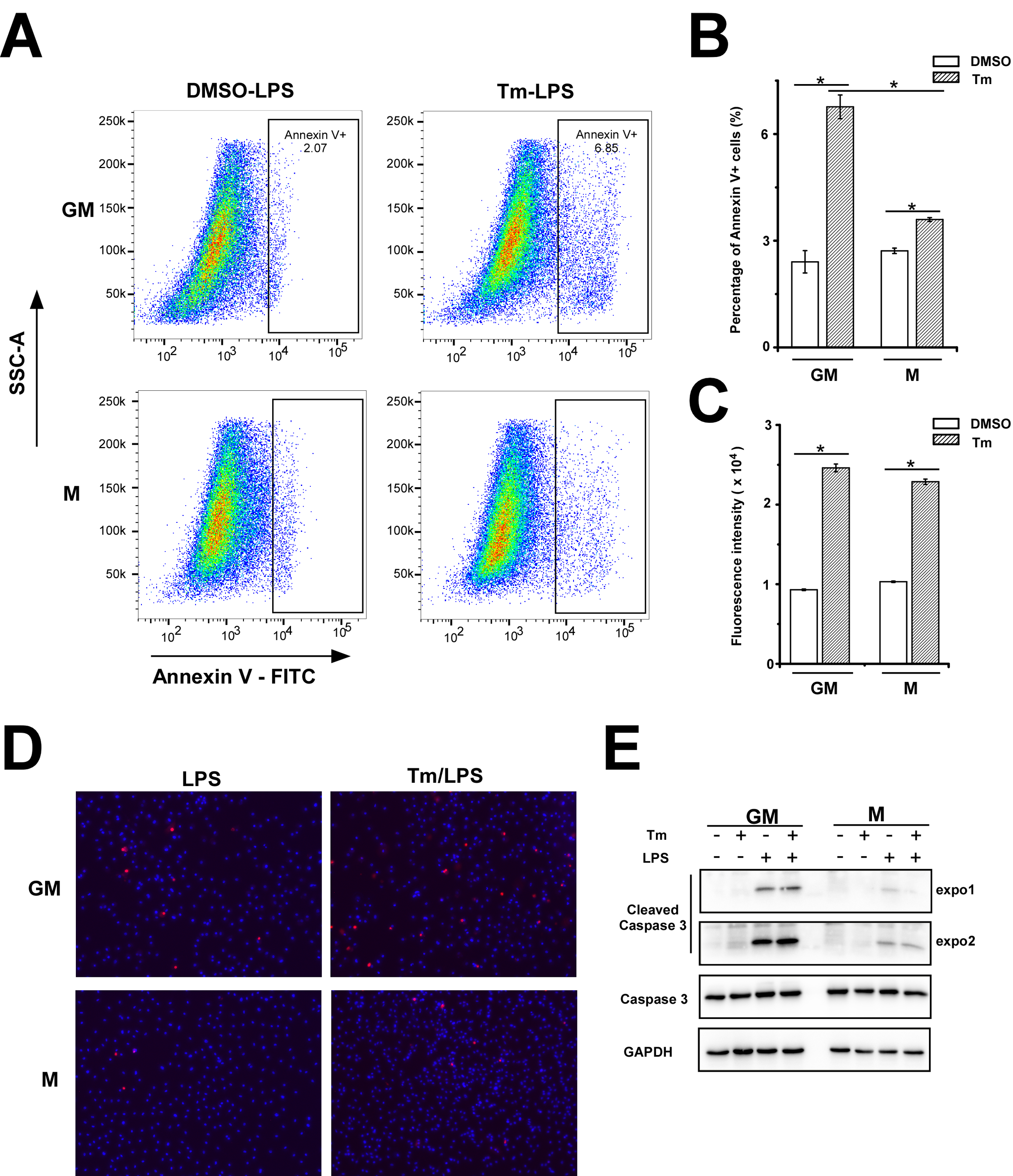

Supplement: Figure S2 — Cellular stress and TLR induced apoptosis in BMDMs. M-BMDM and GM-BMDM were treated with DMSO or Tm (1 mg/ml) for 6 h prior to stimulation with LPS (100 ng/ml) for 10 h. (A) Cells were stained for Annexin and analyzed by flow cytometry. The percentage of annexin V positive cells (B) and the mean fluorescence intensity (C) for each treatment group were quantified. (D) The cells were also stained with PI (red) and Hochest (blue). (E) Levels of cleaved caspase 3 protein from these cells were analyzed by Western blot. Data are presented as the mean ± SD of triplicate experiments and the differences between indicated treatments were evaluated by two way-ANOVA and Turkey's multiple comparisons test. P < 0.05 is indicated by * for comparison of the indicated groups. [file Image_2.TIF]

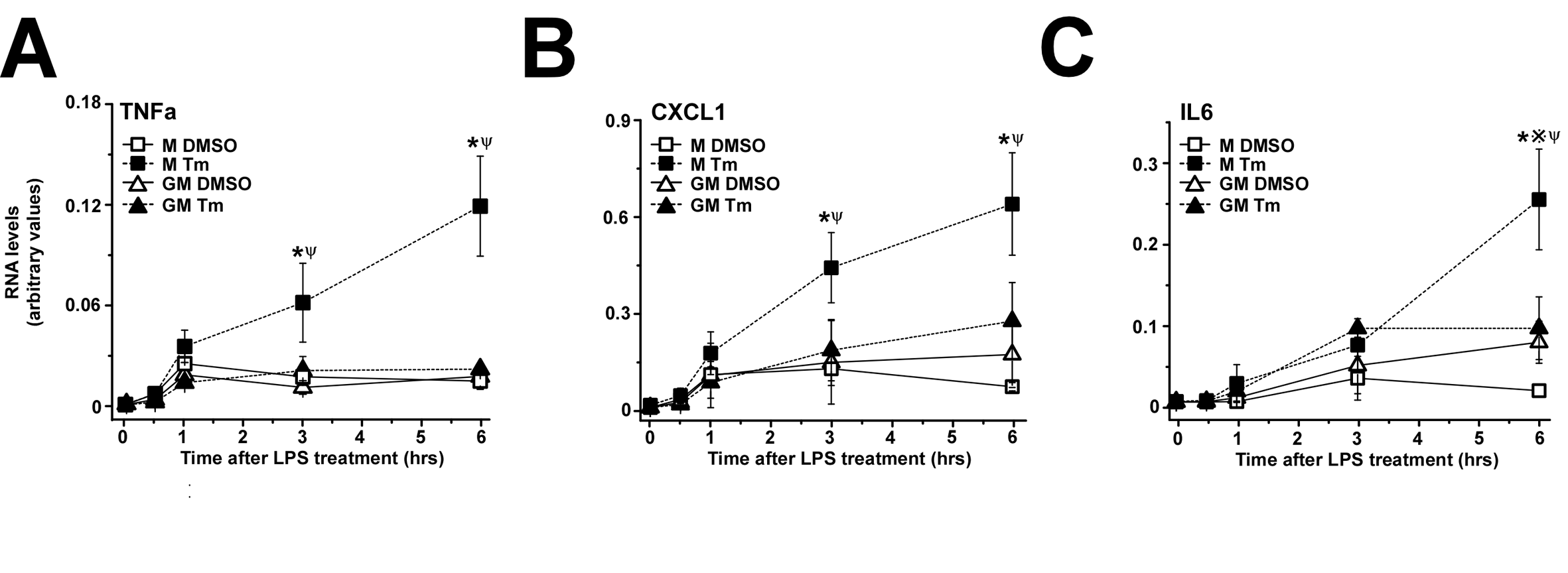

Supplement: Figure S3 — Cellular stress amplifies TLR4 induce cytokine expression in BMDM. (A–C) M-BMDM and GM-BMDM were treated with DMSO or Tm (1 μg/ml) for 6 h prior to stimulation with LPS (100 ng/ml) for the indicated times. Levels of TNFα (A), CXCL1 (B), or IL6 (C) mRNA were determined by real time PCR and presented as described in Materials and Methods. Data are presented as the mean ± SD for triplicate experiments and the differences between DMSO and Tm treatments were evaluated by two way-ANOVA and Turkey's multiple comparisons test. P < 0.05 is indicated by * for comparison of stress vs. DMSO in M-BMDMs, by # for comparison of stress vs. DMSO in GM-BMDMs, by ※ for comparison of M DMSO vs. GM DMSO and by Ψ for comparison of M-BMDM stress vs. GM-BMDM DMSO. [file Image_3.TIF]

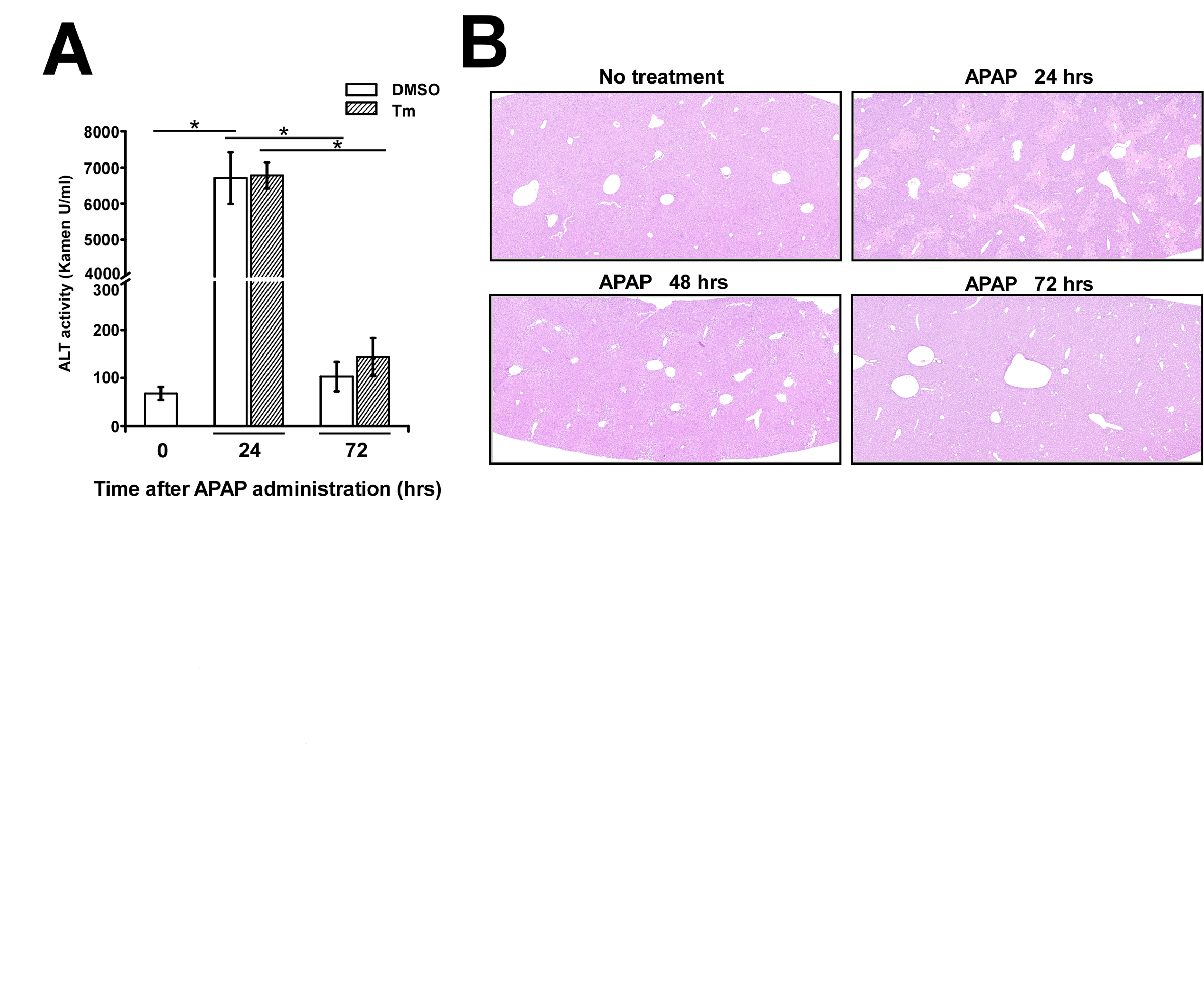

Supplement: Figure S4 — Liver injury induced by APAP administration. (A) WT mice were injected i.p. with APAP (300 mg/kg) for 24 or 72 h and treated with DMSO or Tm i.p. during the final 18 h. The blood was collected for the measurement of ALT activity as described in Materials and Methods. (B) WT mice were treated with APAP only as in (A), and the representative images of H&E-stained liver sections 24, 48, and 72 h post APAP challenge are shown (n = 5). Data are presented as the mean ± SD of triplicate experiments and the differences between indicated treatments were evaluated by two way-ANOVA and Turkey's multiple comparisons test. P < 0.05 is indicated by * for comparison of the indicated groups. [file Image_4.TIF]
